# Thumb reconstruction - down memory lane

**DOI:** 10.1186/1753-6561-9-S3-A8

**Published:** 2015-05-19

**Authors:** Bhaskaranand Kumar

**Affiliations:** 1 Division of Hand and Microsurgery, Kasturba Medical College, Manipal, 576104 India

## 

The importance of the thumb can be gauged from ancient Indian mythology where it is mentioned in the Mahabharatha. Drona poignantly demands the thumb as a gift for his gurudakshina from Ekalavya. As mentioned by Napier, the absence of the thumb, whether congenital or post-traumatic, significantly impairs hand function. Although technical advances have led to changes in surgical methods, the general surgical philosophies of thumb reconstruction have remained relatively constant since their inception. A brief review of the evolution of thumb reconstruction outside of microsurgical techniques is outlined and we have practiced these techniques for the past 25 years. The available procedures within a flexible protocol helps guide the clinician in planning thumb reconstruction. We follow an algorithm where we target restoration of length first followed by stability and finally mobility. The results of various standard options of thumb reconstruction, technical tricks, tips and the pitfalls are shown.

